# “I’m Going to Stop Myself Before Someone Stops Me”: Complicating Narratives of Volitional Substance Use Treatment

**DOI:** 10.3389/fsoc.2021.619677

**Published:** 2021-02-10

**Authors:** David Frank, Suzan M. Walters

**Affiliations:** Rory Meyers College of Nursing, New York University, New York, NY, United States

**Keywords:** methadone maintenance treatment, patient agency, treatment decisions, stigmatized populations, criminalization

## Abstract

**Background:** Often people assume that entry into drug treatment is a voluntary action for persons who use drugs (PWUD). This narrative informs the organizational and regulatory structure of most treatment programs and consequently affects patients’ ability to exert agency over their own treatment. Yet, this view ignores the complex interplay between individual and structural factors in peoples’ decision-making processes, particularly among people who use drugs who are stigmatized and criminalized. Treatment programs that assume voluntary entry may lack appropriate services for the populations of treatment seekers that they serve.

**Methods:** This paper uses semi-structured interviews with 42 participants in Opioid Substitution Treatment (OST) (including patients, clinic doctors and staff, and advocates) informed by one of the author’s own lived experience in OST, to examine patients’ treatment decisions, and in particular, if and how, the structural context of drugs’ illegality/criminalization affected their willingness to pursue treatment. A Critical Discourse Analysis was used to identify key themes.

**Results:** Interview data demonstrates that most people who use drugs enter treatment under constrained conditions related to drugs’ illegality. Themes that emerged included: 1. A feeling of limited choices due to drugs’ illegality; 2. Peer and family pressure; 3. Fear of losing children; and 4. Internalized stigma (i.e. feeling they are dirty or bad for using).

**Conclusion:** Narratives that frame PWUD’s treatment decisions as volitional provide political cover to policies that criminalize PWUD by obscuring their effect on PWUD’s treatment decisions. Treatment models, particularly those that serve highly criminalized populations, should be re-conceptualized outside of normative narratives of individual choice, and be broadened to understand how larger structures constrain choices. By looking at macro-level factors, including the interplay of criminalization and drug treatment, programs can begin to understand the complexity of PWUD motivations to enter drug treatment. Recognizing the role of the War on Drugs as a force of oppression for people who use drugs, and that their treatment decisions are made within that setting, may enable people in treatment, and providers, to develop more productive ways of interacting with one another. Additionally, this may lead to better retention in treatment programs.

## Introduction

Entry into drug treatment is usually conceptualized as a voluntary, unconstrained action taken by people who use drugs (PWUD) and intended to rectify (i.e., treat) the problem of “addiction.” This is evident not only through clinic descriptions that specifically state that their services are voluntary (University Hospitals, 2020), but also through the Substance Abuse and Mental Health Services (SAMHSA)’s Federal Guidelines for Opioid Treatment Programs which states that clinic physicians must receive “voluntary, written, program-specific informed consent to treatment” before patients can be medicated (SAMHSA, 2015: 24). The Recovery Oriented Systems of Care model adopted by SAMHSA in particular relies on presenting MMT as a “voluntary, self-directed, ongoing process” (2015: 39). Yet, this view may ignore the role of larger structural forces such as criminalization and the War on Drugs, in the lives, and treatment decisions, of PWUD.

Although research demonstrates that many people become involved with methadone maintenance treatment (MMT) as a way of avoiding harm associated with illegal substance use, rather than substance use itself, this view is rarely part of how treatment is institutionally conceptualized or organized (Frank 2018; Frank, 2020). Similarly, stigma–both from family, friends, and co-workers, as well as internalized stigma by patients themselves, also play a prominent, and potentially coercive, role in peoples’ decision to enter MMT (Woo et al., 2017; Paquette et al., 2018).

Since most treatment providers conceptualize patients’ decisions to enter treatment strictly through individually focused models, they may lack an appropriate understanding of their patients’ needs, and thus be less equipped to serve them. For example, since most clinics see their patients strictly as “addicts” with a medical/moral problem in need of fixing, they often employ a top-down, and often punitive approach aimed at changing patient behaviors and beliefs rather than providing services designed to reduce harm in their lives (Frank, 2020).

In response, this article uses qualitative data to examine if, and how, OST patients’ descriptions of treatment uptake evince constraint in their decisions. We focus in particular on how the following themes: 1. A feeling of limited choices due to drugs’ illegality; 2. Peer and family pressure; 3. Fear of losing children; 4. Internalized stigma (i.e. feeling they are dirty or bad for using) complicate notions of patient agency and volition in their treatment decisions. Lastly, we argue that by acknowledging such macro-level factors and how they interact with treatment decisions, programs can better organize their services to meet the complex set of issues their patients are facing.

## Background

MMT has been extensively studied for more than 50 years and is considered to be the “gold standard” for reducing substance use as well as many of the harms associated with illegal substance use (Fareed et al, 2009; Schilling et al., 2016; Joseph et al., 2000). Studies have demonstrated its association with reduced rates of crime; overdose, arrest, and transmission of disease (Bell and Zador, 2000; Shi et al., 2007), as well as enabling individuals to live more stable and less risk-involved lifestyles (Joseph et al., 2000; Ball and Ross, 2012). The number of people on MMT has increased from approximately 227,000 in 2003 to over 350,000 in 2015 (Alderks, 2017).

Despite its many benefits, MMT is generally unpopular with PWUD as demonstrated by its consistently low rates of retention (SAMHSA, 2017). According to the United States treatment Episode Data Set, the median length of stay in Medication Assisted Opioid Therapy, generally considered to be a maintenance-model to be used indefinitely, is only 100 days (SAMHSA, 2017). Many have argued that this is partly because MMT, in the United States, is over-regulated, punitive, and organized according an abstinence-only philosophy which is at odds with the needs of many of those using it (Joseph et al., 2000; Harris and McElrath, 2012; Strike et al., 2013). For example, scholars have suggested that a large portion of the patient population are using the treatment, at least in part, as way of reducing the harms of active substance use, caused mainly from the illegality of drug use, rather than as a means to become abstinent (Koester et al., 1999; Harris and Rhodes, 2013; Frank 2018; Frank, 2020). Conceptualizing problematic substance use through the lens of “addiction” has also been criticized by many scholars (Hart, 2017; Fraser et al, 2014; Keane, 2002; Reinarman, 2005), however this narrative is not only dominant culturally, but also informs the organizational and regulatory structure of most treatment programs in the United States (SAMHSA, 2016; White and Mojer-Torres, 2010).

Thus, there is substantial evidence to complicate overly simplified notions of patient volition in regard to substance use treatment and to justify further investigation into this important aspect of treatment. Gaining a better understanding about patients’ motives and experiences with MMT is essential to maximize the benefits of MMT and reach larger numbers of PWUD.

## Methods

This article is based on two years of qualitative research originally conducted by Frank and including both semi-structured interviews and 2 years of ethnographic observations (Reeves et al., 2008). All data has been anonymized and participants are referred to by pseudonyms. All participants provided informed consent and the study was approved by the City University of New York, The Graduate Center Institutional Review Board.

### Recruitment

Frank recruited participants using a combination of purposive and snowball sampling based initially on contacts maintained through his previous experience as a PWUD and as someone on MMT (Panacek and Thompson, 2007). He had used illegal opioids regularly from approximately 1994–2005 in multiple cities including Chicago, IL, New York, NY, Sheffield, United Kingdom, and Portland, OR, and has been in MMT since then at two separate clinics: one in Chicago, IL and one in The Bronx, NY. In some cases, participants were sought out specifically for their unique perspective on the study’s themes, for example, individuals with expertize on the harm reduction resources in New York City. In other cases, participants were recruited in the process of ethnographic observations or through social networks at harm reduction locations. These participants often recommended friends and/or acquaintances to participate. Attempts were also made to recruit participants from a variety of racial, ethnic, and gender groups, however, because of the often-spontaneous nature of the interviews we were not able to collect accurate demographic information for all of the participants. In some cases, participants were already friends and acquaintances who Frank remained in touch with following the study. This is also how he became aware of the more recent circumstances in the participant, Jenny’s, life. Participant recruitment was stopped at 42 because saturation was reached at that point.

### Data Collection

Data was collected in New York City from 2014 through 2016. Frank conducted semi-structured interviews with 42 stakeholders including patients (individuals who had been on MMT for at least 1 year), treatment providers (including Clinic Directors, doctors, counselors), and substance use/treatment advocates (people who were involved with organizations that advocate for people on MMT such as the National Alliance for Medication Assisted Recovery or for the rights of people that use drugs such as the International Network of People that Use Drugs). Interviews lasted approximately 1 h, and were recorded and transcribed by Frank later.

Although interview questions varied by participant category, they each addressed participants’ experiences with, and views of, illegal drug use and treatment. The following domains were addressed: motivations for participation in MMT (either their own motivations or their perceptions of others in the case of treatment providers); recovery (what does it mean to participants; how should it be conceptualized in MMT); clinic organization (rules and regulations; focus on abstinence versus harm reduction); substance use treatment (how well does treatment meet the needs of participants; how should it be organized). Interviews, particularly with MMT patients, tended to be highly unstructured, often taking the form of a dialogue. This was important for two related reasons. First, people on MMT are by definition a marginalized group who are used to exercising caution in regards to what types of information they disclose. For example, admitting to using illegal substances or otherwise acting in ways outside of institutionally accepted behavior can result in serious penalties including dismissal from their clinic. This meant that part of the conversations, particularly early on, involved Frank’s having to gain participants’ trust.

In most cases, Frank revealed his own status as a person on MMT to participants. Although he had initially planned to not reveal any personal information, it quickly became apparent that the benefits of disclosure, in terms of richness and quality of data, as well as the increased honesty and comfort of the study participants, far outweighed the benefits of not “biasing” the data. For example, participants often visibly relaxed or verbally expressed relief upon Frank’s disclosure. Similarly, the familiarity with terminology, common culture, and shared experiences, also helped to position him as part of the community rather than an outsider, who are often (and with good reason) viewed with suspicion.

Secondly, because ideologies of oppression are often internalized (Gorelick, 1991; DeVault, 1996; Reinarman, 2005)—particularly in an institutional setting like MMT (Goffman, 1968; Foucault, 1972; Harris and McElrath, 2012) - it is likely that participants from this group may initially describe their experiences through the institutionally accepted narrative, regardless of how well it aligns with their experiences and/or treatment goals. The dialogue interview format helped to create an environment where participants felt more comfortable describing their experiences in ways outside of those concepts and language.

These types of methodological concerns, which necessarily address internalized power structures and the ideologies that support them, have often been discussed in Marxist and Feminist research (Powers, 2001; Bloom, 1998). For example, numerous feminist scholars, particularly those working with qualitative methods, have rejected the notion of a distanced and neutral observer, choosing a situated approach to knowledge instead (Haraway, 1988; DeVault, 1996). Situated approaches are those that acknowledge the positionality and power relationships existing between researcher, subject, and participant. They are most often used when studying groups that are structurally and/or ideologically marginalized, and generally place a greater emphasis on transparency and reflexivity than on neutrality and objectivity. Situated approaches to research are also more comfortable with the political and activist concerns of research, in that challenging power is seen as a valuable part of the process (DeVault, 1996). Addressing these tensions, feminist scholar Marjorie L. DeVault writes that situated approaches “provide the outline for a possible alternative to the distanced, distorting, and dispassionately objective procedures of much social research.” (1996; p. 29).

Frank also made ethnographic observations in New York City methadone clinics and harm reduction organizations for approximately 2–4 h a week for a period of approximately 6 months. During observations, Frank engaged in discussion with various individuals and assessed the general atmosphere of each location. After each observation period, Frank made notes that were later used to develop study themes.

### Data Analysis

Data was originally coded by Frank for themes using AtlasTi, a software package used for qualitative data analysis. Later on, when the two co-authors agreed to pursue this research question, data was then analyzed by both authors in an iterative process informed by previous literature as well as themes that emerged throughout the research process. Themes included: the role of a substances’ legality/illegality in people that use drugs’ treatment choices; the role of stigma in people that use drugs’ treatment choices; and fear of disrupting family relationship in people that use drugs’ treatment choices; as well as others. Authors met regularly (by phone and Zoom) to discuss the study’s primary themes and focus.

In line-with Frank’s situated approach to data collection, the authors utilize a Critical Discourse Analysis (CDA) approach to the analysis of the data (Fairclough and Wodak. 1997; Fairclough, 2013). CDA, an approach often used in Foucauldian-inspired work, utilizes narratives deployed by different stakeholders, as means of revealing hidden power structures, oftentimes in order to problematize dominant cultural and/or institutional theoretical models of behavior (Van Dijk, 1993; Cook, 2005). In “Discourse Analysis and the Critical Use of Foucault,” Linda Graham describes Foucauldian forms of discourse analysis by their concern with understanding power, representation, and a reticence to see method as an objective means of uncovering “truth” (Graham 2005). She writes that such an analysis would focus on “constitutive and disciplinary properties of discursive practices within socio-political relations of power” as a way of illuminating “how language works not only to produce meaning but also particular kinds of objects and subjects upon whom and through which particular relations of power are realized” (Graham, 2005: 4).

In practice, this meant that both authors discussed the interview texts with an aim to uncover and describe how they fit within larger systems of power, such as drug prohibition and the War on Drugs. Since the two authors occupy different positions (gender, ethnic, personal history) in relation to the subject matter, we used this as a check against leaning too far into either of our positional biases and often discussed the data from a variety of perspectives.

## Results

### Limited Choices due to Drugs’ Illegality

The illegality of heroin (and illegally used prescription opioids) structured and affected the lives of participants and in particular, their decisions about treatment. Nearly all participants focused, to some extent, on how the illegality of heroin affected their decision to attend MMT.

In some cases, this consisted of formal pressure exerted on participants by the Criminal Justice System (CJS). For example, courts sometimes gave participants a simple choice between jail or treatment. Participants described their experiences with courts as confusing, and many were unsure exactly what they were agreeing to. They simply knew that they were avoiding incarceration. For example, Foster, a black man in his early forties, who has been on MMT twice, describes how he became involved with MMT in this way:

“Basically, I felt that I was being chained. At the time, beginning with the courts, [they] had made me get on the program, to do their protocol … I had to get on it [MMT] or else go to jail … Between that with parole, the courts and all that, all that combined in one. So I was forced on it. So I really didn’t really like it at the time, didn’t understand it anyway.” (Foster, 2014)

Here Foster describes a situation where he felt he had little choice, and the overarching goal was to avoid jail. He said that he was “made” and forced into treatment, connoting a lack of free will and agency. He also mentions that he did not understand what he was agreeing to, eluding to another erasure of free will. Involvement with the CJS for most participants, like Foster, was experienced as overbearing, threatening, and confusing and often gave participants the feeling of having little control over their own situations. Literature on the CJS has noted the use of such techniques as a form of social control (Clancey and Howard, 2006; Tiger, 2013).

Participants involved with the CJS also described using treatment as a strategic means of avoiding more severe forms of punishment. For example, some individuals utilized treatment as a way of demonstrating their desire to abstain from drug abuse to judges in order to avoid jail. Thus, even those who didn’t describe their experience as “forced” still describe a context of constraint that significantly reduced their agency and made this kind of legal maneuvering necessary. For example, Monica, a white woman in her 30 s describes her experience like this:

“I wasn’t forced [but] I had legal issues, I was incarcerated for like 28 days and basically was put into a 28-day program since I had never done any treatment programs before … ….Basically the judge, I was in jail a week, and they were like, “If we get her in a program, she can leave right now.” ……..But of course no one is in a hurry. I’m in a hurry, I’m like, “Get me in a program now!” But I can’t call anybody, whatever. So anyway, I just ended up sitting there until somebody decided that they had a place for me to go. And basically, somehow I made the methadone clinic seem a little bit more than what it really was and the judge was like, “Wonderful,” and he considered that outpatient, he overlooked the fact that I was taking Methadone Maintenance Therapy. I was like, “I go to groups, I see my counselor once a week.” I played it up, I sold it and that was fine. Everybody was like, “How did you get methadone maintenance as an outpatient?” I’m like, It worked. So basically, I did have to do an outpatient and they dismissed the charges, everything. I had two felonies and three misdemeanors and I plead out to disorderly conduct.” (Monica, 2014)

Monica’s account also demonstrates how participants evinced agency in the face of constraint. She recounts skillfully convincing a judge to dismiss her charges and even reports having exaggerated the role she believed methadone would play in her life to get an outcome that she preferred. Thus, despite the many forces of constraint that she describes, for Monica, this was a form of ascertaining her agency, and a way for her to be in control of her life.

However, participants did not always become involved with treatment as a result of direct institutional pressure. Many described indirect pressure because of a constant risk from law enforcement that made holding a job, going to school, or establishing a stable life extremely difficult. Others described their reasons for pursuing treatment as being “sick of the hustle” or by simply expressing the desire to never go to jail again.

For example, the next time that Foster was in treatment, he describes how pressures associated with opioids illegality–such as the need to steal in order to support his habit and the consequences that could result—pushed him toward MMT.

David: So the second time that you got on the clinic, that was not court mandated?

Foster: No, no, [I got on MMT that time] cause I was waking up sick too much, and you know, I didn’t want to steal to support my habit. You know that we have to do things to support our habit. So I was on verge of saying, you know what, I’m going to stop myself before someone stops me. (Foster, 2014)

Thus, despite technically entering MMT on his own accord, Foster described his decision as constrained by a framework of structural risks due both to the illegality of opioids themselves, and the need to engage in illegal activities to generate enough funds to purchase them.

Moreover, some participants’ responses suggested that their decision to attend MMT was related to their desire to obtain opioids without the hassles, risks, and legal problems associated with criminalization rather than an attempt to “treat their addiction” or become “abstinent.” For example, Allison described how the constant cycle of craving and withdrawal–an everyday experience for many people that use illegal opioids–prevented her from living a “normal” life. As she describes.

And so, I didn’t want to crave it anymore. And when I found methadone, my thing is I wanted maintenance. I did not want to detox anymore. I just don’t want to crave, because I know for me to detox is not the answer. The whole idea was to stop craving ... so that I would have energy to lead a normal life. Because fighting the crave took too much energy out of my day … Too much energy. I don’t want to fight a crave anymore, and I found that methadone completely alleviates the crave, the thought of it, the desire for it or anything. It just it really limits the crave and for me to detox and be clean there’s always going to be a little bit ... (Allison, 2014)

Thus, in contrast to the dominant institutional narrative that imagines all patients attend MMT as a means of seeking treatment for their addiction, our data shows that for many, it is the access to safe, regulated, and legal opioids that MMT provides which drives many patients there.

### Peer and Family Pressure

Participants also described substantial pressure associated with their relationships with friends and family. Research demonstrates the importance of family and the desire to please them, especially those from marginalized and/or stigmatized populations, in peoples’ choices (Elizur and Ziv, 2001; Paul and Nadkarni, 2017). This sometimes manifested not only in a desire to please people they loved, but also through the complexities of trying to manage a family and related responsibilities while also managing one’s physical dependence to opioids.

For example, in addition to the legal issues she explains, Monica also describes trying to manage related family problems, that were exacerbated by the consequences of her substance use. She states:

“Yeah. So within, I would say, eight months or so or using, I lost my job, because I was a medical assistant for 16 years and I stole copays because the money was, I needed it because I had five kids and my full time job is paying that, they’re in hockey, Catholic school, everything…

So, I had to support my habit. Where was I? So within that eight month period of starting, I lost my kids, my house. My example husband kicked me out, he’s like, “Get out, you’re done,” or whatever. You know, technically I still have custody of them but they live with him. That’s a whole other. And so all of that happening just made my use get worse…

I spiraled. My parents don’t talk to me, don’t talk to us. You’re done. And I was like, this whole unconditional love thing, you’re always here for each other … And it was like, I didn’t get the memo. “We’re always here for you, but if you become a drug addict, that’s it.” So, losing my kids, my parents, my family, it just made it more out of control. And then of course now with no job to support my habit, you start stealing, and that’s where the petty larcenies and stuff came in. *So basically, I was forced into an outpatient. And then afterwards, when I’m in the outpatient they’re like, I said, “I’m thinking about going to methadone.”* (Monica, 2014)

Like many people who use illegal drugs, Monica’s difficulties were exacerbated when her family, who perceived Monica’s problems as caused by her poor individual choices, gave up and began to distance themselves from her. As a result, she felt that she had no other choice but to attend treatment.

The approval of family members and friends also exerted a strong influence on participants’ decision-making regarding treatment. For example, Charles, a white man in his late twenties, described himself as completely unwilling to attend treatment until his girlfriend’s overdose and death led to a dialogue with his father that resulted in his acceptance him of his parents’ desire for him to attend treatment. He states:

Charles: About 5 days later [after the overdose death of his girlfriend], I was sitting drinking Heinekens with my dad watching a soccer game, a European soccer game. My mom was at work and I said to him, I said to my dad, I said, “Dad, I’ve been looking at these methadone clinics, and I think I need to go to one. Can you take me to one after the game?” And he said, “Yeah, I’ll take you.” He’s like, “Your mom’s going to fucking kill me, though.” I said, “Yeah, yeah, I know. I know she’s going to fucking kill you”

David: Because of what? Because she had bad feelings about methadone?

Charles: No, no, because I admitted to them that I had been using heroin for the last 2 weeks, and at that point, I was kind of hooked again.

Thus, family exerted influence over participants both as a coercive force that pressured some participants into treatment, but also through an internalized desire on some to please their family friends by making choices they would approve of.

### Fear of Losing One’s Children

The threat of state intervention, and particularly the potential of Child Protective Services (CPS) to remove children, was a strong motivator for participants, especially women, with children. CPS has extensive powers to make demands over parents they believe to be unfit—particularly when drug use is involved (Johnson and Sullivan, 2008; Choate and Engstrom, 2014). As the following participant describes:

Monica: I went with the National Association of Pregnant Women to the convention in Tennessee—it revolved around pregnancy, drug use, and motherhood. Because they have that law where [if you’re using illegal opioids] they charge you with a felony, I think it’s called the Fetal Assault Law, they’re hoping to change it in July … Because what they’re finding is women crossing state lines to give birth; women not getting prenatal care. There was one women she wasn’t wearing her seatbelt and she saw the cop was gonna pull her over and she just sped away because she knew she was done, she had a warrant, and she was just like, “They’re taking your child away.” Even being on methadone, they consider that being on drugs. And then when you hear the Obstetricians and all these professionals talking [about], you know, being on opioids, or being on methadone, is not as harmful to the fetus, a Xanax, and anti-depressants too.” (Monica, 2014)

As Monica explains, women who are pregnant and use opioids may have their children taken away due to the Tennessee Fetal Assault law which research confirms did lead to an increase in out-of-state births particularly among racial and ethnic minorities (Choi and Leslie, 2020). However, she was surprised when she went to a medical conference and discovered that methadone, anti-anxiety medications, and anti-depressants are all safe to use when pregnant. Despite the science, Monica and others faced real consequences if they used drugs.

The fear of ones’ children being taken away not only affected peoples’ choice to attend treatment but also factored into their choice of which kind of treatment to attend. Specifically, participants sought out treatment models that would model appear more impressive to agencies with the ability to exercise power over their families. Sometimes this meant that women would get off methadone and opt for a less stigmatized drug such as buprenorphine, which they could acquire at a pharmacy once a month.

For example, one participant chose buprenorphine despite her preference for methadone, with disastrous results. Jenny, a 45-year old (at the time of the interview), white women with two children, one of them, a young girl with special needs and significant health issues, stated not only that she preferred methadone because of its greater pharmacological effect toward reducing cravings, but also that she believed it to be better researched and thus felt more comfortable using it, particularly after the birth of her daughter.

“The only time I was on methadone maintenance was, it started when I first found out that I was pregnant with Sandra, I had been on Suboxone, yeah, the Subutex maintenance for a long time, But when Dawn was born with a heart defect and then she had just been diagnosed with autism, at that time my thought was that methadone had a lot more research, and I actually didn’t really have a doctor. So my thought at the time was the best thing to do was go be under their care because I knew after Sandra was born, I knew I was going to have to deal with CPS and all that stuff because I’ve had to with all my kids. But my main thing was safety, I knew that there was research on the methadone, so that was my motivation to switch to the methadone.” (Jenny, 2014)

However, during the pregnancy and birth of her second child, Jenny experienced significant harassment and abuse by medical professionals over her use of MMT. As she describes:

“I was told by that lady [the nurse], “how dare you give that baby that milk,” after the doctor had just been like “please pump milk and give it to the baby.” [She went on saying] “How dare you give that to your baby? Why are you on such a high dose of methadone?.” I said that I didn’t realize I was on a high or low dose—I was on the dose that the doctor gave me. So, long story short, because this story still makes me sick to my stomach, they got to the point because I was on methadone, even though I was in a program, they had this emergency meeting where they were gonna remove Dawn and Sandra from my care.” (Jenny, 2014)

Jenny was able to avoid losing her children, which she believes was only because she and her husband had retained their own therapist, outside of the court system, who was able to speak on their behalf. However, the experience had badly shaken both parents, and she decided to switch to buprenorphine, a similar, though much less stigmatized medication which can be obtained at a pharmacy rather than a methadone clinic. She emphatically stated:

“When I think of how close I came. And the things that these CPS workers, who are supposed to be educated. And the way the nurses treated me … ” (Jenny, 2014)

Unfortunately, since buprenorphine is a partial agonist compared to methadone, a full agonist, as she suspected, it did not prevent cravings as well, and she eventually began injecting to increase its euphoric/therapeutic value. Since she viewed her actions as her own “poor choices” rather than the result of structural and institutional policies, she hid this practice from family, friends, and her doctor. After a few years, the injection site became infected and grew increasingly worse as she continued to inject there. Eventually, she was rushed to the hospital with a dangerously high fever and rapidly deteriorated, falling into a comatose state. A week later she had died from complications associated with clotting and infection of the injection site.

### Internalized Stigma and Societal Stigma

Not surprisingly, stigma against people who use illegal opioids also motivated some participants to use MMT. For example, participants stated that they went into treatment because they didn’t want to remain a “Dopehead” or “fuck-up” any longer. In contrast, when participants where on treatment they referred to themselves as “clean.” By far, this was the most prominent way that self-stigma, also referred to as internalized stigma, manifested.

Many also reported feeling self-conscious about how they looked and were perceived by others. Participants reported feeling as though others perceived them as dirty or mistrust worthy. For example, one participant, a white man in his late twenties stated:

“Back when I was using, I looked like a piece of shit. I mean, I could’t even walk into a regular store without a cashier being like, “Oh, here’s a fucking junkie.” And that was, I’ll say, after I got off it, I’d been clean about 76 days, going back, I was like, “I don’t want to look like that again. I don’t want to have myself perceived like that again.” (Charles, 2014)

Although this is complicated by the fact that MMT is also stigmatized, since methadone is legal, it is far easier to hide and manage compared to heroin which must be obtained through illicit and unreliable sources often multiple times a day. As such, participants perceived MMT to be the better option because it was legal and less stigmatizing than illicit drug use. Yet, oftentimes, they could not quite shake the stigma, which manifested as internalized stigma.

In line with this view, Foster did not conceptualize his use of MMT through narratives of treatment of recovery but saw it as a way of dealing with the contextual realities of illegal substance use. For example, the following conversation demonstrates this:

David: Okay. Do you consider yourself as being in recovery now? Now that you’re on methadone?

Foster: No.

David Frank: No. Tell me why.

Foster: Because I know deep down I’m not really *clean*….I’m just doing something to maintain.

Here Foster uses the language of “clean” to describe someone who does not use drugs and delineate such individuals from himself. In line with many 12 step programs ideologies, methadone is indeed considered a drug, and therefore someone using it is not drug free, or in Foster’s words “clean.” Ideas such as this were prominent among participants in this study.

## Discussion

This article examines if, and how, OST patients’ descriptions of treatment uptake evince larger forms of constraint. Findings demonstrate that patients’ treatment decisions are often made within a context of constraint that limits their agency. Similarly, narratives that position OST patients’ treatment decisions as strictly volitional ignore the role of larger, structural forces in the lives of people who use drugs. Instead, we argue for an approach to understanding peoples’ treatment decisions that better reflects MMT’s position within complex, external, and often oppressive, structural contexts that drive people who use illegal drugs to treatment.

Although the decision to enter substance use treatment, or a particular type of substance use treatment, is typically conceptualized as an unconstrained action, like all social phenomenon, it is the result of a complex interaction between individual and structural forces (Mills, 2000). These forces are not discrete but rather interact with, and reinforce each other, pushing people who use drugs into particular treatment decisions. Our data demonstrates that in contrast to the institutionally dominant view which describes treatment in purely volitional terms, external forces, experienced as coercive, played a substantial role in participants’ treatment choices. In particular, participants experienced pressure related to: 1. A feeling of limited choices due to drugs’ illegality; 2. Peer and family pressure; 3. Fear of losing children; and 4. Internalized and societal stigma (i.e. feeling they are dirty or bad for using). However, within situations of constrained choice participants often still found ways to assert their agency. For example, those who were able to use MMT to their benefit, especially as a strategy to avoid incarceration.

All of the themes that emerged were directly influenced by larger structural forces that were out of the control of participants, mainly the illegality of drug use which carried with it the threat of incarceration and/or losing one’s children. Policies criminalizing drugs likely not only affect individual choices, such as choosing between treatment or incarceration, but also affect family and friends perceptions of drug use and well as one’s perception of self (i.e., internalized stigma). Further, people who use drugs do not live in isolation, they have family and social networks whom they care about, and whom they would like to please and keep in their lives. Thus, the consequences of criminalizing drugs influenced treatment decisions for participants, not only directly to avoid criminalization, but also to please family and friends and to gain a better self-worth (though not always achieved completely).

Therefore, narratives that position peoples’ treatment choices as purely volitional are problematic, firstly, because they misrepresent the needs of PWUD. As Frank has argued previously, if treatment is conceptualized individually, without acknowledging its role as a refuge from criminalization, it is likely to embrace a punitive model in-line with that discourse’s focus on the need for individual change (Frank, 2018). Moreover, such policies provide political cover to policies that criminalize PWUD. By framing peoples’ decisions to enter treatment as unconstrained, individually based choices, the coercive harm created by policies like criminalization and the War on Drugs, in the lives of PWUD is erased in favor of a narrative based strictly on sick/bad people choosing to “get better.”

This analysis aligns with the work of a growing body of multi-disciplinary research that is critical of the nearly universal use of “addiction” to understand substance use and treatment (Frank, 2018; Fraser et al., 2014; Campbell, 2012). Although addiction-as-disease models still dominate both in scholarly and lay settings (Volkow and Fowler, 2000; Volkow et al., 2016), scholars have been increasingly critical of its lack of conceptual clarity and rigor and focus solely on the individual as an agent of harm (Keane, 2002; Reinarman, 2005; Davies, 2013; Fraser et al., 2014). For example, social scientists, like Suzanne Fraser and Nancy Campbell, have questioned how well-suited the concept of addiction is to understand Medication assisted Treatment (MAT) (Fraser and Valentine, 2008; Campbell, 2011). Similarly, Rebecca Tiger’s work on Drug Courts demonstrates that such interventions, which are based on an addiction-as-disease view of substance use, can cause more problems than they solve (2013).

It also aligns with the many critiques of MMT as being overly punitive. For example, researchers have pointed out problems with MMT’s restrictive take-home policies, intrusive use of drug testing, and a power differential between patient and provider that almost certainly contributes to low rate of use and retention (Frank, 2020; Strike and Rufo, 2010; Damon et al., 2017; Pani and Pirastu, 2000). Evincing this, low-threshold clinics, that aim to reduce such barriers, demonstrate better rates of patient retention and satisfaction as well as reducing harms such as overdose mortality and all-cause mortality (Nolan et al., 2015; Strike et al., 2013;

It is important to point out that this analysis focuses specifically on MMT, a treatment model whereby patients remain using opioids. While the authors believe that criminalization and the War on Drugs exert pressure on PWUD to enter all forms of treatment, it is likely to be strongest in Opioid Substitution Treatment models, like MMT, because of this fact.

There are several limitations to this research. Firstly, that one of the two authors is on MMT could be considered a source of bias (the other author is not, which could also be considered a bias). However, research using Marxist, feminist, and other post-structuralist-inspired theoretical methods such as CDA, often accept that all positionality is biased, and distinctions made within scholarly work between bias and objective or insider vs. outsider are artificial (Fairclough and Wodak, 1997; Fairclough, 2013). Yet, we do not think that such distinctions are meaningless toward an interpretation of our data, and thus, we are being transparent about Frank’s use of both illegal heroin and MMT. Similarly, as this study is not based on a representative sample, results cannot be generalized to a larger population of PWUD. Moreover, we were also unable to collect accurate demographic information for all of the study participants, partially because many of the interviews began informally, through conversation. Additionally, since this research was conducted in New York City participants likely had better access to MMT than in less urban geographic areas. Research has demonstrated the dearth of services for people who use illegal drugs in non-urban settings (Jones, 2018; Cochran et al., 2019). Similarly, because of the clustering of harm reduction services in urban locations, participation in MMT is probably less stigmatizing than in other settings. In light of that, results may not be transferable to less urban locations. However, we could potentially conclude that PWUD in less urban parts of the US likely experience even more coercion and negative consequences for using substances.

Nevertheless, this research has important implications for how drug treatment is conceptualized and administered.

We argue that narratives which conceptualize individuals’ decision to attend treatment as strictly a matter of individual choice are reductive and problematic by ignoring the tremendous socio-political pressures, primarily due to drugs’ illegality, and related problems, on peoples’ decisions regarding substance use treatment. Rather, the analysis of such decisions should be broadened to include an understanding of how larger structural forces—notably criminalization and the War on Drugs—constrain the agency of people who use illegal drugs in all of their decisions, but especially those related to treatment. Yet importantly, they do not mute the agency of people using drugs, and many people find ways within incredibly constrained conditions to navigate their trajectories as they feel is most beneficial for them (Koester et al., 1999; Mateu-Gelabert et al., 2010; Harris and Rhodes, 2013). Acknowledging the interplay between individual and structural forces in the treatment decisions of criminalized drug users, and how a person’s agency is constrained due to these forces, will not only provide a more sophisticated and evidence-based understanding of PWUD’s motivations, but can also provide a more productive platform from which to identify criminalization and the War on Drugs as forces of harm in the lives of people using illegal drugs. Moreover, it may pave the way for new approaches to treatment so that we can meet the United States goals of providing substance use treatment to a greater number of people (Healthy people, 2020).

## Data Availability

The raw data supporting the conclusions of this article will be made available by the authors, without undue reservation.
